# Supplementary material for: Extracellular Cysteines Are Critical to Form Functional Cx46 Hemichannels
Source: Int J Mol Sci. 2022 Jun 29;23(13):7252. doi: 10.3390/ijms23137252 (PMC9266770; doi:10.3390/ijms23137252)
Supplement: Supplementary file 1 [file ijms-23-07252-s001.zip › ijms-1743156-supplementary.pdf]

## Supplementary material

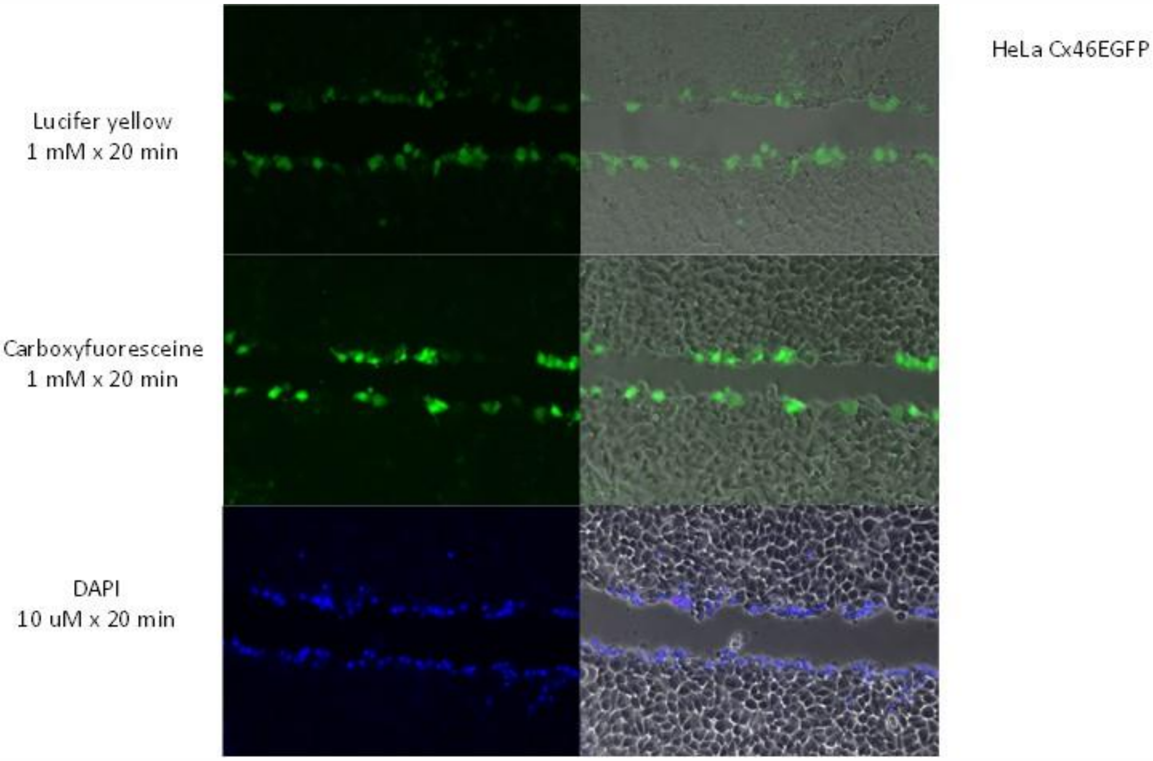


**Supplementary Figure S1.** To assess whether transfection of HeLa cells with Cx46 WT (Cx46-EGFP) induces the presence of functional gap junction channels (GJCs) we performed a scrape loading experiment using Lucifer yellow (upper), carboxyfluorescein (CF, middle) and DAPI (bottom). After 20 min of exposure to the dyes, no dye transfer between HeLa cells expressing Cx46-EGFP was observed for any of the dyes used. Left: fluorescence; right: fluorescence and transmitted light.

## Method

Gap junction permeability was determined at room temperature using the scrape-loading/dye transfer (SL/DT) technique. HeLa cells transfected with Cx46-EGFP were plated and incubated with either Lucifer yellow (1 mM), carboxyfluorescin (500 µM) or DAPI (10 µM) in recording media, followed by scrape-loading, which was performed with a razor blade. After 20 min, the plates were washed with recording media without dye, and examined by epifluorescence.

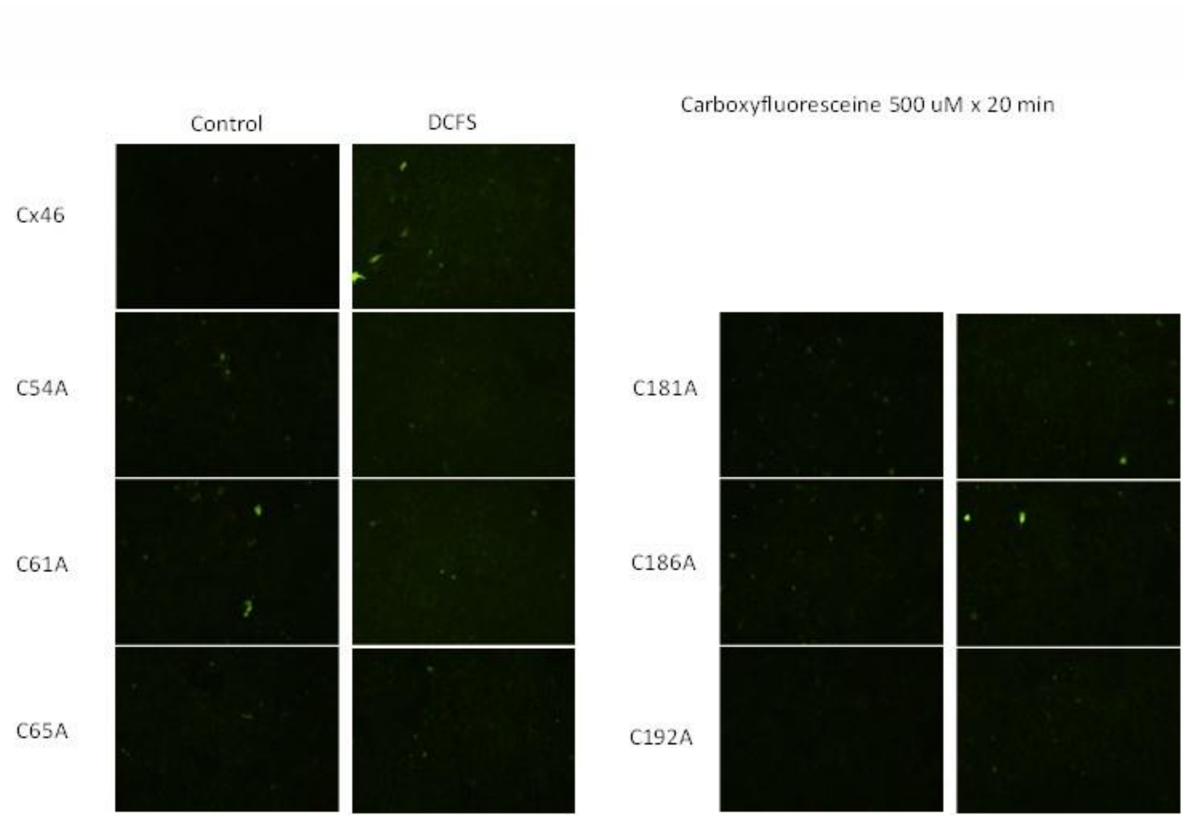


**Supplementary Figure S2.** Carboxyfluorescein (CF) uptake in Cx46 WT (Cx46) and Cys mutants. HeLa cells transfected either with Cx46 WT or Cys mutants showed very low permeability to CF under normal (control) conditions and in DCFS.

Method

HeLa cells were placed in an epifluorescence inverted microscope (Nikon, Ti-Eclipse) adapted for time-lapse studies and were exposed to an external solution containing 500 $\mu$M carboxyfluorescein in DCFS, which consisted of 140 mM NaCl, 4 mM KCl, 2 mM $CaCl_2$, 5 mM glucose, 5 mM EGTA, and 10 mM Hepes, at pH 7.4. After 20 min, the cells washed extensively with recording media containing $Ca^{2+}$ and $Mg^{2+}$ and pictures were taken.
